# The Effect of Implant Length and Diameter on Stress Distribution around Single Implant Placement in 3D Posterior Mandibular FE Model Directly Constructed Form In Vivo CT

**DOI:** 10.3390/ma14237344

**Published:** 2021-11-30

**Authors:** Akikazu Shinya, Yoshiki Ishida, Daisuke Miura, Akiyoshi Shinya

**Affiliations:** 1 Department of Dental Materials Science, School of Life Dentistry at Tokyo, The Nippon Dental University, 1-9-20 Fujimi, Chiyoda-ku, Tokyo 102-0071, Japan; yishida@tky.ndu.ac.jp (Y.I.); daisuke@tky.ndu.ac.jp (D.M.); 2 Department of Prosthetic Dentistry and Biomaterials Science, Institute of Dentistry, University of Turku, Lemminkaisenkatu 2, FI-20520 Turku, Finland; 3 School of Life Dentistry at Tokyo, The Nippon Dental University, 1-9-20 Fujimi, Chiyoda-ku, Tokyo 102-0071, Japan; shinya-a@tky.ndu.ac.jp

**Keywords:** short implant, stress distribution, finite element method, implant length, implant diameter

## Abstract

A three-dimensional (3D) finite element (FE) model of the mandibular bone was created from 3D X-ray CT scan images of a live human subject. Simulating the clinical situation of implant therapy at the mandibular first molar, virtual extraction of the tooth was performed at the 3D FE mandibular model, and 12 different implant diameters and lengths were virtually inserted in order to carry out a mechanical analysis. (1) High stress concentration was found at the surfaces of the buccal and lingual peri-implant bone adjacent to the sides of the neck in all the implants. (2) The greatest stress value was approximately 6.0 MPa with implant diameter of 3.8 mm, approx. 4.5 MPa with implant diameter of 4.3 mm, and approx. 3.2 MPa with implant diameter of 6.0 mm. (3) The stress on the peri-implant bone was found to decrease with increasing length and mainly in diameter of the implant.

## 1. Introduction

Dental implants are prosthetic devices which restore masticatory function or improve aesthetic appearance. A metal post is surgically implanted in the bone at an area of a missing tooth or external trauma, and this acts as the support for a superstructure. In many cases of tooth deficiency with minimal intervention, implants have been selected due to favorable and reliable results, with a 10-year survival rate of over 95% [1,2,3,4].

For long term function of implants, there should be no bone resorption in response to the repeated force exerted through mastication, and for the patient to maintain physiologically sound periodontal tissues. Furthermore, mechanical problems may occur at the bone–implant interface in implants that are subjected to occlusal force [5], and there has been increasing interest in mechanical research into the implant–bone interface. Moreover, implants that have osseointegrated lack the presence of the periodontal ligament in natural teeth, which functions to absorb impact at the contact area between the bone and implant. As a result, unlike natural teeth, the load is transmitted directly to the bone. Consequently, implants with bone remodeling are affected by bone morphology and quality to a greater extent than natural teeth [6,7]. For this reason, ensuring a favorable prognosis with implant therapy requires not just biological examination, but also the resolution of mechanical problems.

Moreover, with a wide range of implants currently in clinical use, it is difficult to select the type of implant best suited to the individual needs of the patient. At present, in the absence of any clear selection criteria for implants, selection is almost always made on the basis of the clinician’s experience with mandibular bone size and previous clinical outcomes. For this reason, three-dimensional visualization techniques using dental X-ray computed tomography (CT) scanning devices have become effective methods of examination for making accurate diagnoses [8,9,10,11]. Three-dimensional X-ray CT devices are able to measure the bone morphology and density of live individuals. It has been reported that these data can be used to produce 3D finite element (FE) models that reproduce with a high degree of precision the detailed morphology of the bone as well as material properties that accurately reflect the internal structure of the bone [12,13,14,15]. Stress analysis calculated by FE method showed an excellent distribution of bone around implant while any forces applied. Some articles provided stress distribution any type of implant [16,17,18]. If it were possible to perform virtual implant therapy simulations using 3D X-ray CT scans of live individuals prior to the actual procedure, treatment planning for implants could be carried out with a high level of mechanical predictability, which could become a new diagnostic standard in clinical practice. This would contribute to highly safe and successful implant therapy, from examination and diagnosis to planning and surgery.

Here, a 3D FE model that reconstructs the bone morphology and material properties of the mandible was created from X-ray CT scans of a live subject. The model was used for mechanical simulations to evaluate the effects of thickness and length of implants on the stress exerted on the peri-implant bone, in order to investigate the usefulness of virtual implant diagnoses in clinical settings.

## 2. Materials and Methods

### 2.1. Three-Dimensional (3D) X-ray CT Scanning

The mandible selected for scanning was that of a healthy 20-year-old male with no caries, who gave his informed consent. The study protocol was approved by the Ethics committee of Nippon Dental University.

A CT scanner (Alphard Series Alphard-3030; Asahi Roentgen, Kyoto, Japan) was used to scan the data needed for the study, and the 3D X-ray CT scanning conditions are shown in Table 1. Four hundred and sixty scanned images were obtained, and these were saved as Digital Imaging and Communications in Medicine (DICOM) files, the standard for medical-use images.

### 2.2. Construction of 3D FE Model from 3D X-ray CT Images

**Figure 1:**
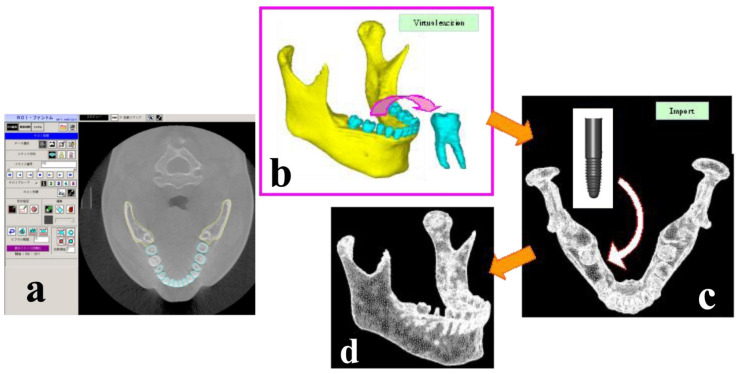
Procedure for the virtual implantation on the mandibular 3D FE model. (**a**): Extracting bone lines, (**b**): 3D FE model, (**c**): Discretized implant, teeth, and mandibular bone model, (**d**): Completed FE model. (Reproduced from Ref. [13]).

Integrated digital image processing and finite element analysis software (Mechanical Finder ver. 5.1; Research Center of Computational Mechanics, Osaka, Japan) were used to reconstruct the 3D FE model on a computer from the data converted to DICOM files. The procedure for image processing and creation of the analysis model is shown in Figure 1. First, of the 460 scans taken over 17 s, 239 images of CT slices corresponding to the mandible, which were used for the 3D FE model in the present study, were selectively input into Mechanical Finder (MF). The number of images varies according to the slice thickness of the scanning conditions, and the thickness used in the present study allowed the creation of an elaborate model with minimum radiation and was therefore safe for the body. Following this, region of interest (ROI) processing was performed on the area of the model to be reconstructed. ROI processing is a method by which threshold processing is performed on the site for direct analysis on CT images in order to extract a region for modeling. The region for modeling is extracted by surrounding the target region. The cross-section images on which ROI processing was performed were combined on a computer, and the 3D FE model was automatically reconstructed by layering the images. Regions where the threshold processing was imperfect because the values were close to the threshold, were corrected manually.

First, the mandibular bone and the teeth were classified separately in order to create a 3D FE model capable of virtual implant therapy simulation. Figure 1a a shows the frontal plane of the skull, Figure 1b shows a CT slice of a horizontal section of the mandible, Figure 1c shows the mandible comprising trabecular and cortical bone, and 1d shows the completed 3D FE model created from 3D X-ray CT images.

### 2.3. Virtual Placement of the Implant

**Figure 2:**
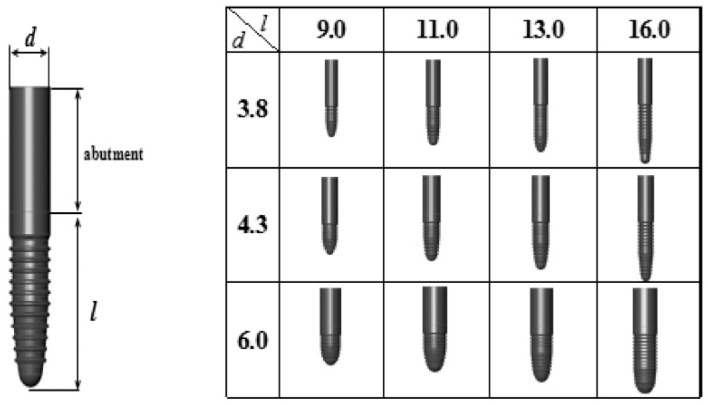
Implant 3D models (d: diameter, l: length, mm).

Virtual extraction of the first molar was performed on the constructed mandibular 3D FE model, and Standard Triangulation Language (STL) data were created for the extraction site by a 3D CAD program (VX-CAD/CAM; Machine Ware, Tokyo, Japan; hereinafter, VX). Three-dimensional FE models with virtual placement of implants of different shapes were then created using the STL data. A total of 12 one-piece implants (Camlog: Alta-Dent, Tokyo, Japan) were used, combining three diameters (D = 3.8, 2.3 mm, and 6.0 mm) and four lengths (L = 9.0 mm, 11.0 mm, 13.0 mm, and 16.0 mm). The shape data of implants with each of the 12 shapes were converted to 3D models on VX and then imported to MF, and 3D FE models of the implants were created by element breakdown. The 3D FE models of the implants are shown in Figure 2. Virtual extraction of the right mandibular first molar was performed on a computer, and virtual placement of the implants was performed by placing each implant of different shape into the extraction socket, in the buccolingual center of the alveolar bone and aligned axially to the teeth.

The space in the mandibular ridge following virtual extraction was set to be automatically replaced by bone, so there was no bone in the space when the implant was placed.

### 2.4. Material Properties and Boundary Conditions

**Figure 3:**
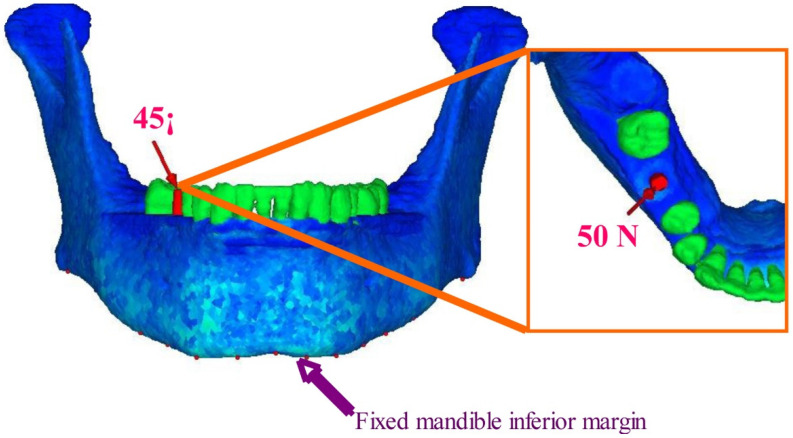
Geometrical and boundary conditions. A buccolingual force at a 45-degree oblique angle to the tooth axis was concentrated onto a single contact point, and static occlusal load of 50 N was applied to the premolar buccal cusp of teeth. Inferior border of the mandible was assumed to be fixed (Reproduced from Ref. [13]).

The material properties of the bone, teeth, and implants are shown in Table 2. The material properties of the implants assume that they are made of titanium, which is the material most widely used in commercially available implants [12]. With regard to the material properties of the mandibular bone, for each individual element the bone density ρ at that point was calculated from the CT values by the formula ρ = ct × 0.945 × 0.0001, and a calculated value of the mean Young’s modulus E was selected according to Keyak’s conversion formula [19,20]. A material constant was thus assigned to each element, and the analysis took the density distribution of the bone into account. ρ is the bone density in gf/cc, CT is the CT value in Hounsfield units (HU), and E is Young’s modulus in MPa. Performing the above conversion operation allowed non-linear material properties that accurately reflected the detailed inner structure of the bone to be recreated. The boundary conditions are shown in Figure 3. With regard to restriction conditions, the inferior border of the mandible was assumed to be completely fixed. The site of the load was defined as a single nodal point on the outer inclination of the buccal cusp, where a concentrated load was applied at an angle of 45° to the axis of the tooth from the buccal side in the lingual direction. The load was set at 50 N, which was assumed to be the average occlusal force of mastication applied to the prosthetic device abutted by the implant. A stress analysis using the finite element method was carried out on the 12 mandibular 3D FE models with virtual implants of different shapes placed following extraction, in order to compare the maximum stress values in the peri-implant bone and examine the stress distribution seen in the peri-implant bone.

The analysis was linear static analysis, the material properties of the bone were set as non-linear material, and the results were evaluated as equivalent stress. The models each had a total of 54,728 nodes and 302, 335 4-node tetrahedral elements. The models were created, and FE analysis was performed on a PC Workstation (Precision Workstation 670, Dell Inc. Round Rock, TX, USA).

## 3. Results

**Figure 4:**
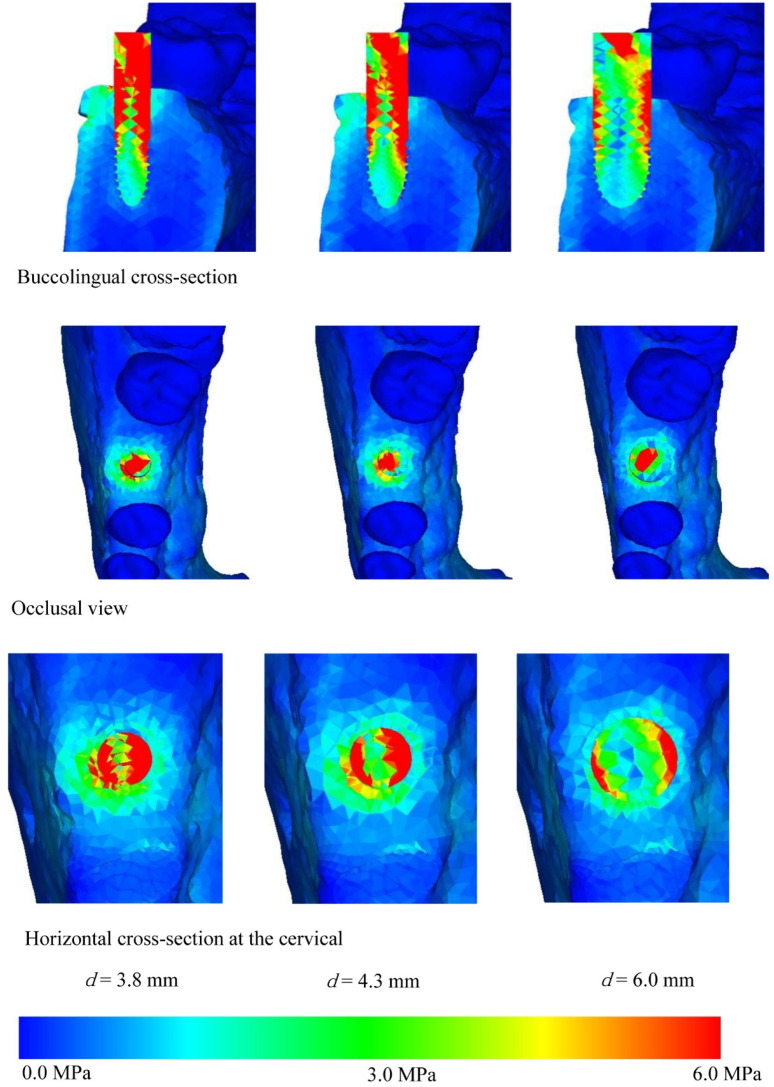
Equivalent stress distribution (*l* = 9.0 mm).

**Figure 5:**
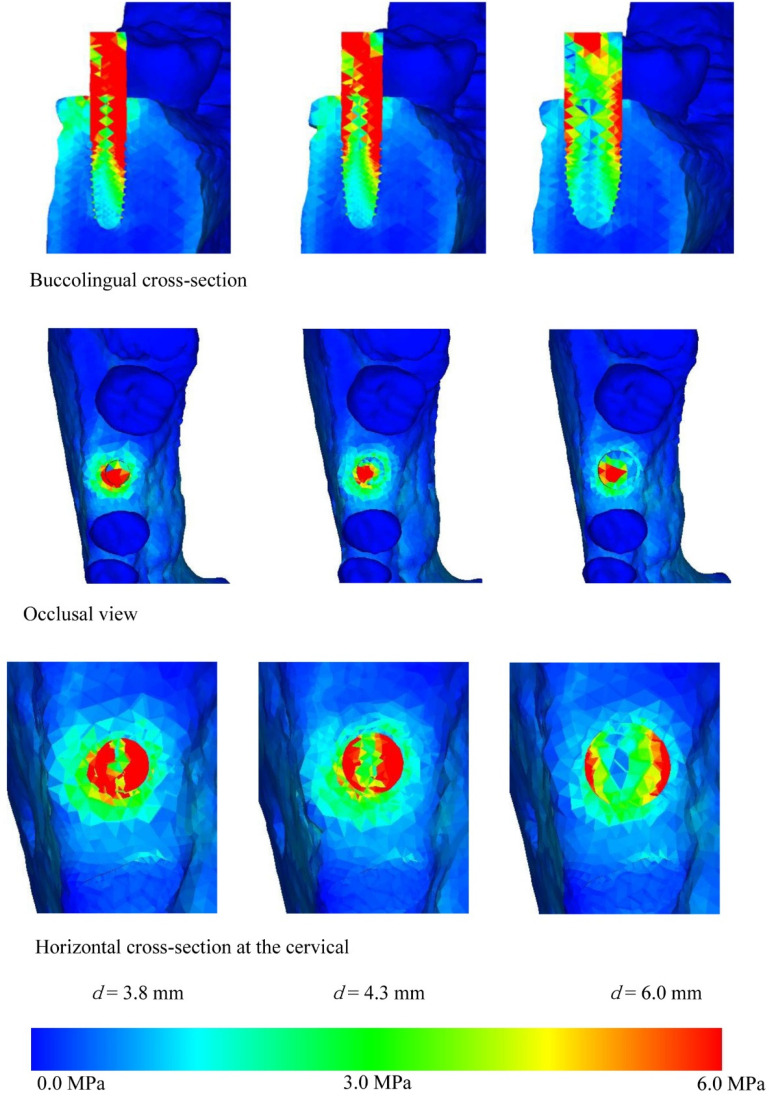
Equivalent stress distribution (*l* = 11.0 mm).

**Figure 6:**
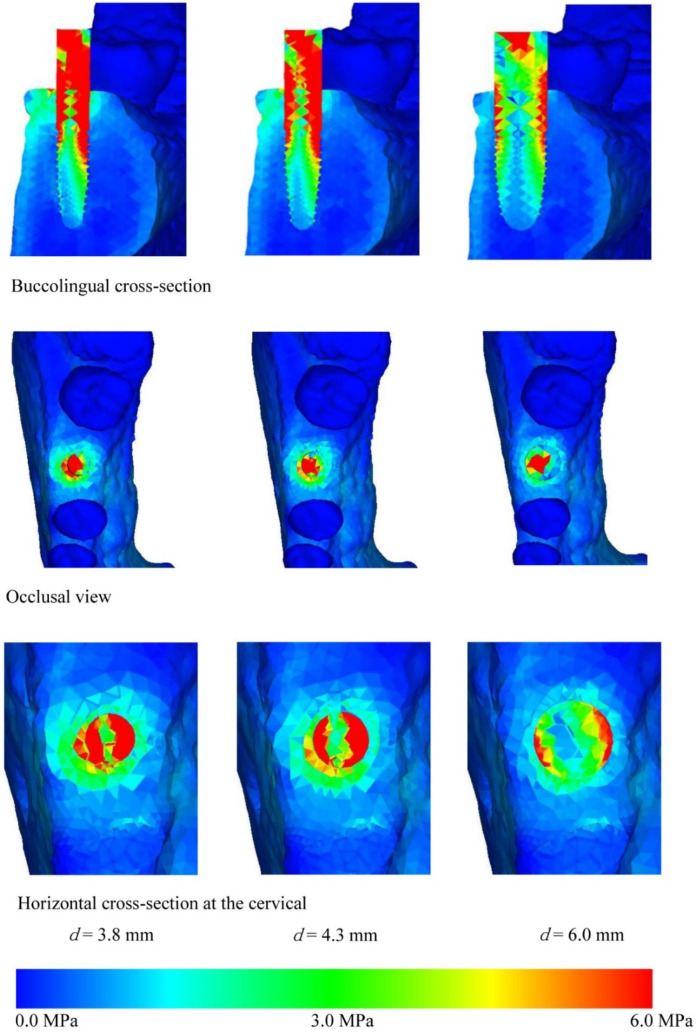
Equivalent stress distribution (*l* = 13.0 mm).

**Figure 7:**
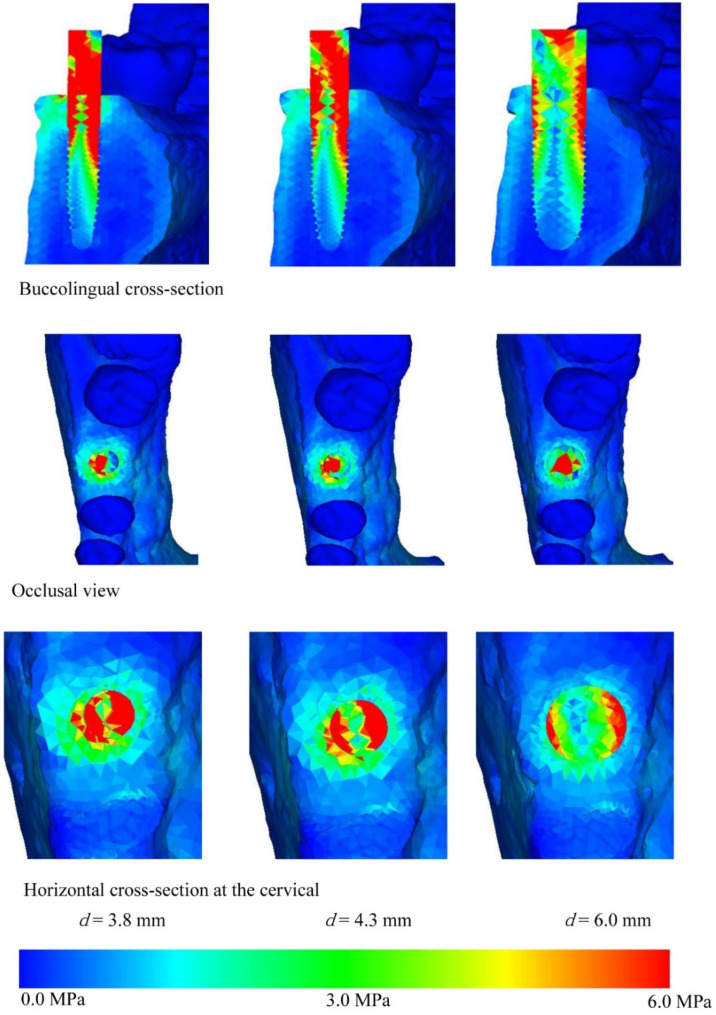
Equivalent stress distribution (*l* = 16.0 mm).

**Figure 8:**
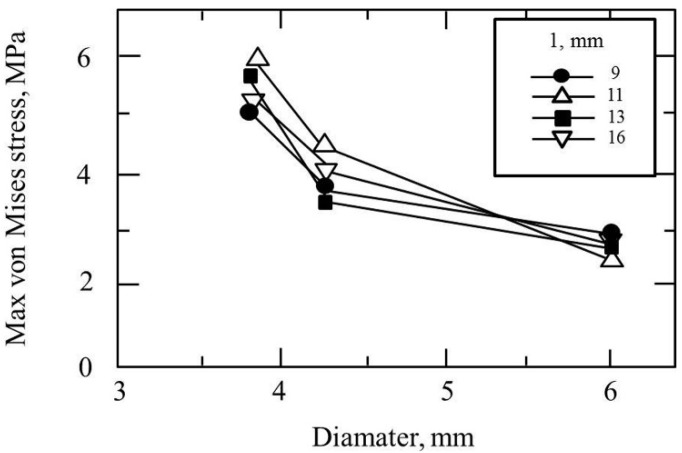
Equivalent stress distribution of implant surrounding bone.

Figure 4, Figure 5, Figure 6 and Figure 7 show the equivalent stress for each implant type in a buccolingual cross-sectional view, occlusal view, and horizontal cross-sectional view at the neck of the implant. In all implants, high distribution of stress was found in the peri-implant bone surface on the buccal and lingual sides in the region of the neck. The maximum stress values for implant lengths of 9.0, 11.0, 13.0, and 16.0 mm were respectively approx. 6.0, 5.8, 5.3, 5.0 MPa for implant diameter 3.8 mm; 4.5, 4.0, 3.8, and 3.5 MPa for implant diameter 4.3 mm; and 3.2, 3.0, 2.8, and 2.5 MPa for implant diameter 6.0 mm. The maximum equivalent stress values in the peri-implant bone are shown in Figure 8. The results show that the equivalent stress values on the peri-implant bone tend to decrease with increasing length and diameter of the implant.

The buccolingual cross-sectional view showed equivalent stress in the peri-implant bone surface on the buccal side. The equivalent stress was approximately 3.0–6.0 MPa with implant diameter of 3.8 mm, 3.0–4.5 MPa with implant diameter of 4.3 mm, and 1.0–3.0 MPa with implant diameter of 6.0 mm. The occlusal view of the implant and the horizontal cross-section at the cervical region show concentration of the stress in the peri-implant bone, and this is particularly high on the buccal side. With the buccolingual cross-section, equivalent stress values tend to decrease in the same way with increasing diameter and length of the implant. In the implant, equivalent stress was found from near the load-bearing site toward the base of the implant, and the equivalent stress decreased with increasing length and diameter of the implant. The stress distribution was such that stress appeared in peri-implant bone but tended to decrease in the central and lower regions.

A 3D FE model is created of the mandible, comprising cortical and trabecular bone from data obtained non-invasively from CT scan images, and the implant, which was designed by CAD. Using this model, virtual implants of different diameter and length are placed in a tooth extraction socket and examined using the finite element method. Mechanical simulations using CT scans, CAD, and FEM in this way were shown to be useful.

## 4. Discussion

### 4.1. Experimental Method

The finite element method is a technique for stress analysis that was developed as computers grew more sophisticated. This technique separates solid bodies, which are continuous, into countable elements. By calculating the mechanical balance of each element, the displacement or stress of the body as a whole can be determined. Although creating the model entails considerable difficulty, the method permits accurate measurement of internal stress and offers considerable versatility in the methods for evaluating the results. In the present study, a mechanical evaluation was performed by stress analysis using the finite element method.

Principal stress and Von Mises stress (equivalent stress) are often used as methods for evaluation of stress in finite element analyses of bodies with complex shapes such as teeth or mandible [21,22]. Principal stress is often used as an index of rupture or fracture of brittle materials, and it can distinguish the orientation and code whether the stress is positive or negative (i.e., contractile or tensile). Von Mises stress is known to be useful as an index not just of ductile materials but also as an index of rupture or fracture of many materials. Equivalent stress was elected for the present analysis in order to allow comparison of stress distribution between different models, and the evaluation results were compared and examined.

A 3D FE model is needed on the computer when a finite element analysis is performed, and this sort of analysis is problematic in terms of efficiency when using conventional methods because of the time and the labor involved in creating a 3D FE model. Furthermore, it is extremely difficult to create 3D FE models of live subjects, and previously it was common to use 3D FE models created from mean anatomical values or dry skulls. However, there is a need in clinical implant practice for the development of diagnostic systems that incorporate mechanical analysis in order to ensure therapy with a high degree of predictability. For this reason, it is essential to be able to create 3D FE models within a short space of time that accurately reflect the living body. X-ray CT devices are currently used for a wide variety of cases, and have become indispensable scanning devices in the field of implant therapy.

Here, we created a 3D FE model directly from 3D X-ray CT images in a short space of time that accurately reflected the inherent bone shape and bone quality of the living body, and we performed virtual implant placement on a computer using 3D FE models of implants of various different shapes created by CAD. We showed that if it were possible in clinical practice to draw up a mechanically optimal therapeutic plan for living individuals in this way, objective predictions of the prognosis can be made from a mechanical perspective, which is not possible with the conventional method of judging bone morphology only from data obtained by visual examination of scans. This could become a revolutionary diagnostic system in the field of dentistry in the future.

### 4.2. Analysis Program

MF is a finite element analysis program capable of creating a 3D FE model from 3D X-ray CT scan data. A major advantage of this program is that the 3D FE model can be moved and parts can be added or deleted at will comparatively easily. This functionality means that MF has wide applicability, not just in the field of research but also in clinical practice [23,24]. Further, the program can calculate the shape and material property data needed to assess bone strength [15,19] from 3D X-ray CT scans, so that highly reliable results can be obtained. Typical finite element analysis programs often lack the functionality to create 3D FE models, so that CAD programs or stand-alone dedicated 3D FE modeling programs are mainly used. With MF, however, 3D FE modeling can be carried out from 3D X-ray CT scans of the body and the non-linear material properties of bone can be calculated using various different formulae, so the program allows analysis on a 3D FE model that reflects the bone quality [25,26].

### 4.3. Stress Analysis Using Implants of Different Diameters and Lengths

Analysis was performed with implants of different combinations of diameter and length. There have been reports that slender implants have weak mechanical strength and short implants give little support to the teeth [20,27,28,29]. The current situation is that in the absence of any clear criteria, selection is almost always made on the basis of the clinician’s experience with factors such as mandibular bone size or previous clinical outcomes [30]. It is therefore extremely important to build up a clear picture of the mechanical effects that differences in diameter or length of implants have on the peri-implant tissues when a static load is applied, as these data can be indices for implant selection. Sotto-Maior et al. reported that the short-wide implants showed higher stress values that were distributed through a higher area directed to the implant apex [31]. In the present study, 12 implants were selected, with a combination of three diameters (D = 3.8 mm, 4.3 mm, and 6.0 mm) and four lengths (L = 9.0 mm, 11.0 mm, 13.0 mm, and 16.0 mm). Three-dimensional FE model implants were created with a 3D CAD program, using an implant of a size frequently used in clinical practice (D = 4.0 mm and L = 10.0 mm) for reference.

Comparing equivalent stress, the stress was concentrated in the neck of the implant and the surface of the peri-implant bone. Nearer to the base of the implant, the stress diminished. Comparing stress values, longer, thicker implants tended to have less stress. However, looking at the stress distribution shown in Figure 4, Figure 5, Figure 6 and Figure 7, the stress of approximately 6.0 MPa distributed inside the implant showed the same distribution trends in all cases. This is probably because when an implant is longer in the direction of the load, the additional length is within the bone and there is therefore little change in shape. Load acting at an oblique angle to the longitudinal axis of the implant may be defined as the net force of a vertical load and a horizontal load. The horizontal load exerts a bending moment on the implant, and the overall behavior of the implant can be envisaged as that of a cantilever. The stress distribution of a cantilever is greatly affected by the cross-sectional thickness of the beam and the distance from the fixed point. Thus, the stress distribution showed a marked decrease in stress with implants of greater diameter because the thicker implants effectively opposed the bending moment. However, when the overall length of the implant is increased, there is little change in the shape of the part of the implant assumed to be a cantilever. This is because any increase in the length of the implant is within the mandibular bone, whereas the length of the implant above the margin of the bone, which is the part on which the bending moment acts, is the same in all models. In clinical practice, if the implant is lengthened, the longer portion is all inside the bone and the length of the implant above the margin of the bone remains the same, so the conditions are the same as in the present study. Therefore, when the load assumed in the present study is exerted on the implant, increasing the thickness of the implant is more effective in reducing stress than increasing the length. This result was obtained with a load in which the horizontal and vertical components were equal, and further analysis of the effects of implant shape on vertical load is needed [32,33,34].

The results of the present study were roughly the same as when maximum principal stress was evaluated, and implant selection criteria suited to the shape and the quality of the bone were shown when virtual implant therapy was carried out on the subject who had been scanned for the study.

## 5. Conclusions

A 3D FE model of a mandibular bone was created from 3D X-ray CT scanned images taken of a live subject. Simulating the clinical situation of implant therapy at the mandibular first molar, virtual extraction of the tooth was performed on the 3D FE mandibular model and 12 different implants diameters and lengths were virtually inserted in order to carry out a mechanical investigation. High stress concentration was found on the surfaces of the buccal and lingual peri-implant bone adjacent to the sides of the neck of all implants. The greatest stress value was approx. 6.0 MPa with implant diameter of 3.8 mm, approx. 4.5 MPa with implant diameter of 4.3 mm, and approx. 3.2 MPa with implant diameter of 6.0 mm. The stress on the peri-implant bone was found to decrease with increasing length and mainly in diameter of the implant.

## Figures and Tables

**Table 1:** Exposure conditions of CT scanner.

Tube voltage	80 kV
Tube current	5 mA
Voxel size	0.39 mm
Exposure time	17 s
Exposure mode	C-mode (Cephalo CT)

**Table 2:** Material properties of mandibular bone, teeth, and implant.

	Young′s Modulus (MPa)		Poisson′s Ratio
Mandibular bone	*p* = 0	E = 0.001	0.4
	0 < *p* ≤ 0.27	E = 33,900 *p*^2.20^	
	0.27 < *p* < 0.6	E = 5507 *p* + 469	
	0.6 ≤ *p*	E = 10,200 *p*^2.01^	
Teeth	4.80 × 10^4^		0.23
Implant (titanium)	11.0 × 10^4^		0.29
	Mandibular bone (Keyak formula)		
